# Temporal activity patterns suggesting niche partitioning of sympatric carnivores in Borneo, Malaysia

**DOI:** 10.1038/s41598-021-99341-6

**Published:** 2021-10-06

**Authors:** Miyabi Nakabayashi, Tomoko Kanamori, Aoi Matsukawa, Joseph Tangah, Augustine Tuuga, Peter T. Malim, Henry Bernard, Abdul Hamid Ahmad, Ikki Matsuda, Goro Hanya

**Affiliations:** 1 Graduate School of Advanced Science and Engineering, Hiroshima University, Higashihiroshima, Hiroshima Japan; 2 Japan Orangutan Research Center, Tokyo, Japan; 3 Primate Research Institute, Kyoto University, Inuyama, Aichi Japan; 4 Wildlife Research Center, Kyoto University, Kyoto, Japan; 5 Forest Research Centre, Sabah Forestry Department, Sandakan, Sabah Malaysia; 6 Sabah Wildlife Department, Kota Kinabalu, Sabah Malaysia; 7 Primate Studies-Borneo, Institute for Tropical Biology and Conservation, Universiti Malaysia Sabah, Kota Kinabalu, Sabah Malaysia; 8 Institute for Tropical Biology and Conservation, Universiti Malaysia Sabah, Kota Kinabalu, Sabah Malaysia; 9 Chubu University Academy of Emerging Sciences, Kasugai, Aichi Japan; 10 Japan Monkey Centre, Inuyama, Aichi Japan

**Keywords:** Biological techniques, Ecology, Evolution, Zoology

## Abstract

To propose proper conservation measures and to elucidate coexistence mechanisms of sympatric carnivore species, we assessed temporal activity patterns of the sympatric carnivore species using 37,379 photos collected for more than 3 years at three study sites in Borneo. We categorized activity patterns of nine carnivore species (one bear, three civets, two felids, one skunk, one mustelid, one linsang) by calculating the photo-capturing proportions at each time period (day, night, twilight). We then evaluated temporal activity overlaps by calculating the overlap coefficients. We identified six nocturnal (three civets, one felid, one skunk, one linsang), two diurnal (one felid, one mustelid), and one cathemeral (bear) species. Temporal activity overlaps were high among the nocturnal species. The two felid species possessing morphological and ecological similarities exhibited clear temporal niche segregation, but the three civet species with similar morphology and ecology did not. Broad dietary breadth may compensate for the high temporal niche overlaps among the nocturnal species. Despite the high species richness of Bornean carnivores, almost half are threatened with extinction. By comparing individual radio-tracking and our data, we propose that a long-term study of at least 2 or 3 years is necessary to understand animals’ temporal activity patterns, especially for sun bears and civets, by camera-trapping and to establish effective protection measures.

## Introduction

Approximately 20% of the world’s mammal species face the risk of extinction, and this rate has become rapid mainly due to threats such as habitat loss and over exploitation^1^. Particularly, the status of mammals in the Indomalayan region is the worst among the world’s biogeographic realms^1^. The leading causes of this status are anthropogenic factors such as hunting, habitat degradation, and invasive species^1^. Regardless of this urgent issue, we still await effective and realistic solutions because of the scarcity of basic ecological information on mammals linking between local communities and conservation biologists in the Indomalayan region^2^. Information regarding the temporal activity patterns of animals is crucial for assessing responses to anthropogenic disturbances and will allow the implementation of proper conservation measures by using the temporal activity information as indicators of disturbance levels^3^. Moreover, understanding the temporal activity patterns of animals may contribute to elucidate their coexistence mechanisms, which is one of the major themes in ecology^4^.

From an ecological perspective, investigating the temporal activity patterns of closely related sympatric species is critically important to understand their coexistence mechanisms in relation to interspecific competition and/or niche separation, especially among species in the same guild. Closely related species usually have similar morphology, physiology, behavior, and ecology, and therefore competition among them, especially inhabiting in the same area is intense^5^. In most cases of closely related sympatric species, to avoid interspecific competition among them, one or more differences in temporal and spatial activity patterns, and/or diet is present^5^. Mammalian carnivores are a typical taxon that adapts to a specific diet, and their internal and external morphologies are suitable for carnivorous diets^6^. Therefore, competitive interactions with food resources may occur among sympatric carnivore species. To reduce the negative effects of competition, such as interspecific killing and to increase effective access to food resources, sympatric carnivore species flexibly change the temporal activity patterns for their temporal niche partitioning between 2 ≤ species with similar body size and/or utilizing similar-sized prey^7–9^. In addition, considering that their temporal activity patterns can change in response to environmental conditions, such as disturbance level^10^, temporal activity patterns should be evaluated at the site level.

Asian rainforests possess a far larger number of sympatric carnivores than other tropical regions, such as Neotropics and Afrotropics^11^. Among the Asian rainforests, carnivore species diversity is reported to be high in Borneo^12^. The taxonomy of some carnivore taxa is still controversial, but for now Borneo has one bear, five felids, four mustelids, four otters, one linsang, at least eight civets, and two mongoose species, of which at least three are endemic^13^. Despite these species-rich communities, almost half of the Bornean carnivore species are threatened with extinction^14^. Some of the Bornean carnivore species are among the apex predators, for example, Sunda clouded leopards (*Neofelis diardi*), in each community^15^. Others are important seed dispersers, such as civets^16,17^; therefore, they have high value in being protected due to being ecologically important key species that maintain their living ecosystem. Nonetheless, the current information is too limited, and sporadic to understand their basic behaviors, such as temporal activity patterns, which may affect the progress in evaluating and improving the threatened status. This could be because many species of terrestrial carnivores are elusive and difficult to detect in general because of their naturally low density^18^.

Camera-trapping is the most effective method for studying cryptic animals, such as carnivores^18^. Consequently, many studies on the temporal activity patterns of Bornean carnivores have been conducted. However, these are mainly based on small sample sizes, collected in one site during limited periods, and focusing only on one or a few species. Although there are several long-term studies on the temporal activity patterns of Bornean carnivores that are based on more than 3 years of camera-trapping efforts^9,19,20^, the total number of photos available for each species in one study is small. Notable exceptions are studies^9,19^ that reported the spatio-temporal interactions among Bornean carnivores by camera-trapping for more than 6 years at seven to ten sites. However, information on the temporal activity patterns of Bornean carnivores is still limited, especially detailed studies of temporal activity overlaps among non-felid carnivores. To evaluate the threatened status and species interactions, it is necessary to assess the temporal activity patterns of multiple species at multiple study sites is inevitable.

In this study, we assessed daily activity patterns of sympatric carnivores using our comprehensive photo dataset, including not only felids but also other carnivore species collected for more than 3 years at three study sites in Sabah, Malaysian Borneo. The objective of this study was to investigate the differences in temporal activity patterns among the study sites and species.

## Results

### Recorded species and preparation for analysis

We captured 1261 photos of carnivores in total, and there were 753, 218, and 290 photos were taken in Danum Valley Conservation Area (DVCA), the Lower Kinabatangan Wildlife Sanctuary (LKWS), and Tabin Wildlife Reserve (TWR), respectively. We recorded one bear, i.e. Sun bear (*Helarctos malayanus*): n = 83, four civets, i.e. banded civets (*Hemigalus derbyanus*): n = 362; binturongs (*Arctictis binturong*): n = 9; common palm civets (*Paradoxurus philippinensis* see Veron et al. 2015): n = 188; Malay civets (*Viverra tangalunga*): n = 410, five felids, i.e. a bay cat (*Catopuma badia*): n = 1; Sunda clouded leopards: n = 3; flat-headed cats (*Prionailurus planiceps*): n = 3; leopard cats (*P. bengalensis*): n = 27; marbled cats (*Pardofelis marmorata*): n = 10, one skunk, i.e. Sunda stink badgers (*Mydaus javanensis*): n = 10, two mustelids, i.e. Malay weasels (*Mustela nudipes*): n = 2, yellow-throated martens (*Martes flavigula*): n = 16, at least two otter species (two of *Aonyx*, *Lutra*, *Lutrogale* spp.): n = 6, one linsang, i.e. banded linsang (*Prionodon linsang*): n = 25, and two mongoose species (*Urva* spp.): n = 87 (Table 1). We excluded records of the bay cat, Sunda clouded leopards, flat-headed cats, binturongs, and Malay weasels due to their small sample size (< 10). We also omitted the data of the mongooses and otters from analyses because of the difficulty in identifying these taxa at the species level based on photos that captured only a part of their body. However, we used *Mongoose* spp. data to fit a circular kernel density.

**Table 1:** The number of independent photo-capture of carnivore species in the three study sites. Numbers in parenthesis indicate the photo-capture frequency (number of independent events/100 camera-trap days).

Family	Species	Number of photos			
		DVCA	LKWS	TWR	total
Urusidae	Sun bear	15 (0.11)	23 (0.13)	45 (0.90)	83
Viverridae	Banded civet	195 (1.38)	12 (0.07)	155 (3.11)	362
	Binturong	7 (0.05)	–	2 (0.04)	9
	Common palm civet	78 (0.55)	85 (0.47)	24 (0.48)	187
	Malay civet	346 (2.45)	26 (0.14)	38 (0.76)	410
Felidae	Bay cat	1 (0.01)	–	–	1
	Flat-headed cat	3 (0.02)	–	–	3
	Leopard cat	16 (0.11)	5 (0.03)	6 (0.12)	27
	Marbled cat	8 (0.06)	–	2 (0.04)	10
	Sunda clouded leopard	2 (0.01)	1 (0.01)	1 (0.02)	4
Mephetidae	Sunda stink badger	3 (0.02)	27 (0.15)	1 (0.02)	31
Mustelidae	Malay weasel	1 (0.01)	1 (0.01)	–	2
	Yellow-throated marten	13 (0.09)	1 (0.01)	2 (0.04)	16
	Otter	6 (0.04)	–	–	6
Prionodontidae	Banded linsang	7 (0.05)	8 (0.04)	10 (0.20)	25
Herpestidae	*Mongoose* spp.	54 (0.38)	29 (0.16)	4 (0.08)	87

**Figure 1:**
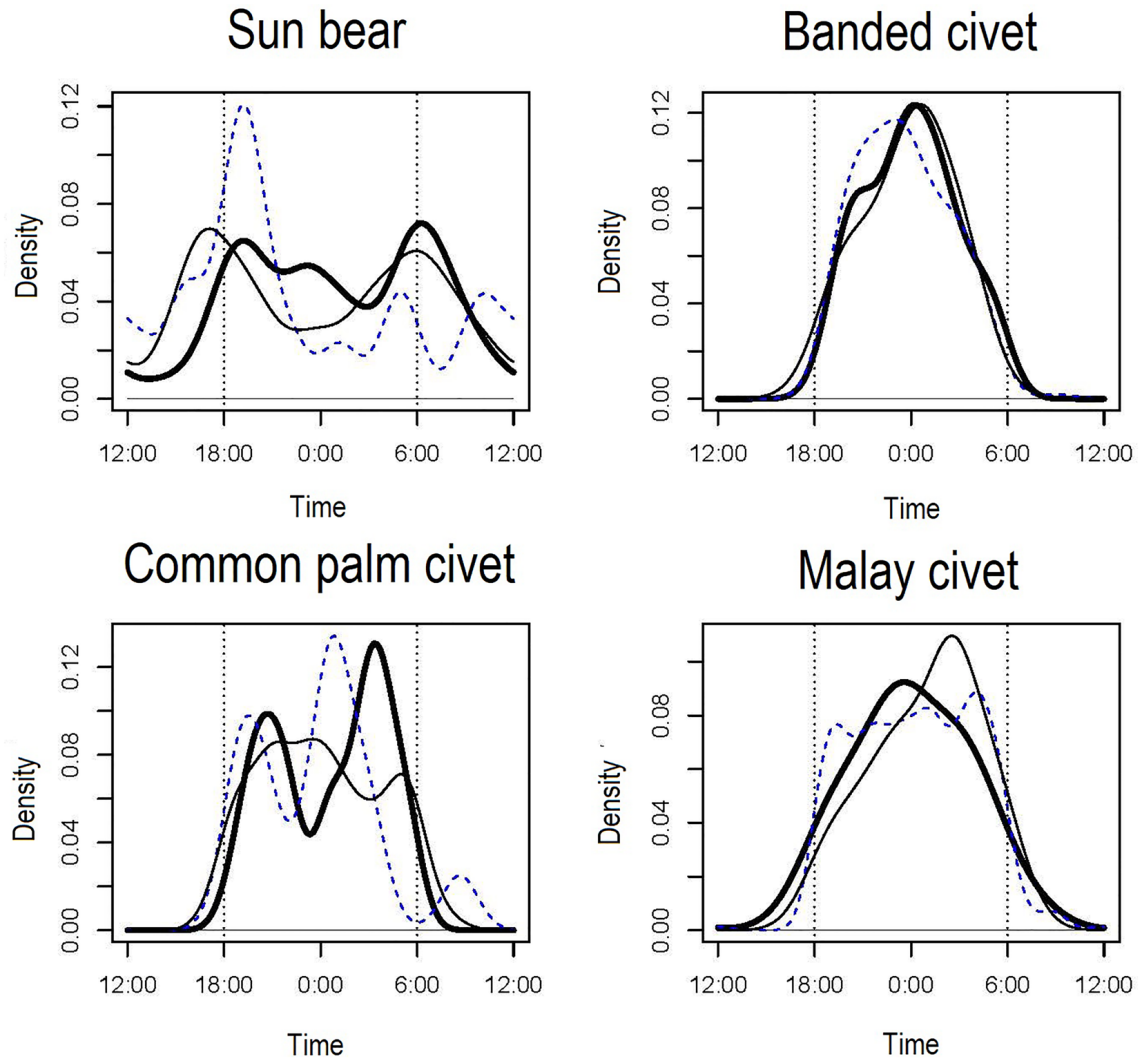
Overlaps of activity patterns of the four carnivore species among the three study sites. Dotted line, solid line, and thick line indicate kernel density estimations in DVCA, LKWS, and TWR, respectively. Dotted vertical lines indicate approximate times of sunset and sunrise, and short vertical lines under the kernel density curves indicate photo-captured times of each species.

The sample sizes of the three civets (banded civets, common palm civets, Malay civets) and sun bears were more than ten in each study site, and we compared their activity levels among the study sites. We found no significant differences in the activity levels of these four species among the study sites (all p > 0.016) (Fig. 1); therefore, we pooled photos taken from the three study sites for all the independent carnivore species records.

### Determination of temporal activity patterns

We applied GLMMs to determine the highest activity period for the carnivore species that were photo-captured more than 50 times: Malay civets, banded civets, common palm civets, and sun bears. Sun bears had no significant differences in the recorded periods (Wald χ^2^ = 3.71, p = 0.16). Banded civets were not recorded during daytime, and were recorded significantly more at night than during twilight (Wald χ^2^ = 6.47, p = 0.01) (Table 2). There were significant differences in the recorded periods in Malay civets (Wald χ^2^ = 18.0, p < 0.01) and common palm civets (Wald χ^2^ = 26.2, p < 0.01). Both of them were more active at night than twilight (z = − 3.46, p < 0.01 in Malay civets; z = − 4.93, p < 0.01 in common palm civets), and there were no differences in the recorded photo numbers between daytime and twilight (z = 1.43, p = 0.31, in Malay civets; z = 0.04, p = 0.99 in common palm civets) (Table 2). Malay civets were more active at night than during the day (z = 3.22, p < 0.01), but the significance was marginal in common palm civets (z = 2.19, p = 0.06), probably due to the small sample size of their daytime activity (n = 3) (Table 2).

**Table 2:** The proportion of independent photo-capture of carnivore species during nighttime, twilight, and daytime.

Family	Species	Night	Dawn	Day	Dusk	Night	Period		
		0000–0459 h	0500–0659 h	0700–1659 h	1700–1859 h	1900–2359 h	Night	Day	Twilight
Urusidae	Sun bear	0.20	0.12	0.29	0.13	0.25	0.45	0.29	0.25
Viverridae	Banded civet	0.42	0.04	0.01	0.05	0.50	**0.92**	0.01	0.09
	Common palm civet	0.38	0.10	0.03	0.09	0.40	**0.78**	0.03	0.18
	Malay civet	0.41	0.09	0.03	0.09	0.38	**0.80**	0.03	0.18
	(Binturong)	–	–	–	–	–	6	3	0
Felidae	Leopard cat	0.33	0.08	0.12	0.14	0.34	**0.67**	0.12	0.22
	Marbled cat	0.05	0.04	0.66	0.10	0.15	0.19	**0.66**	0.15
	(Bay cat)	–	–	–	–	–	0	1	0
	(Flat-headed cat)	–	–	–	–	–	2	0	1
	(Sunda clouded leopard)	–	–	–	–	–	2	0	2
Mephetidae	Sunda stink badger	0.46	0.07	0.05	0.05	0.38	**0.84**	0.05	0.12
Mustelidae	Yellow-throated marten	0.02	0.09	0.65	0.19	0.04	0.05	**0.65**	0.28
	(Malay weasel)	–	–	–	–	–	0	1	1
	(*Otter* spp.)	–	–	–	–	–	0	5	1
Prionodontidae	Banded linsang	0.43	0.20	0.05	0.02	0.29	**0.73**	0.05	0.22
Herpestidae	(*Mongoose* spp.)	–	–	–	–	–	0	68	19

Data of species in parenthesis indicate the number of independent photo-capture. All the data are derived from pooled photo-captures of the three study sites. Values in bold letters indicate the significantly most active period of each species.

For major species that were photo-captured less than 50 times; Sunda stink badgers, leopard cats, banded linsangs, yellow-throated martens, and marbled cats, we tested the selectivity of the active period. We found that the proportion of active periods differed significantly in all five species (χ^2^ = 109.0, p < 0.01 in Sunda stink badgers; χ^2^ = 4.92, p < 0.01 in leopard cats; χ^2^ = 78.0, p < 0.01, in banded linsangs; χ^2^ = 6.10, p < 0.01 in yellow-throated martens; χ^2^ = 4.44, p < 0.01 in marbled cats). Sunda stink badgers, leopard cats, and banded linsangs used nighttime more than expected (p < 0.01) and less than expected during day time (p < 0.01) and twilight (p < 0.01) (Table 2). Yellow-throated martens used daytime more than expected (p < 0.01) and nighttime less than expected (p < 0.01), and there were no significant differences in the usage between daytime and twilight (p = 0.28) (Table 2). Marbled cats used daytime more than expected (p < 0.01) and less than expected at nighttime (p < 0.01) and twilight (p < 0.01) (Table 2).

**Figure 2:**
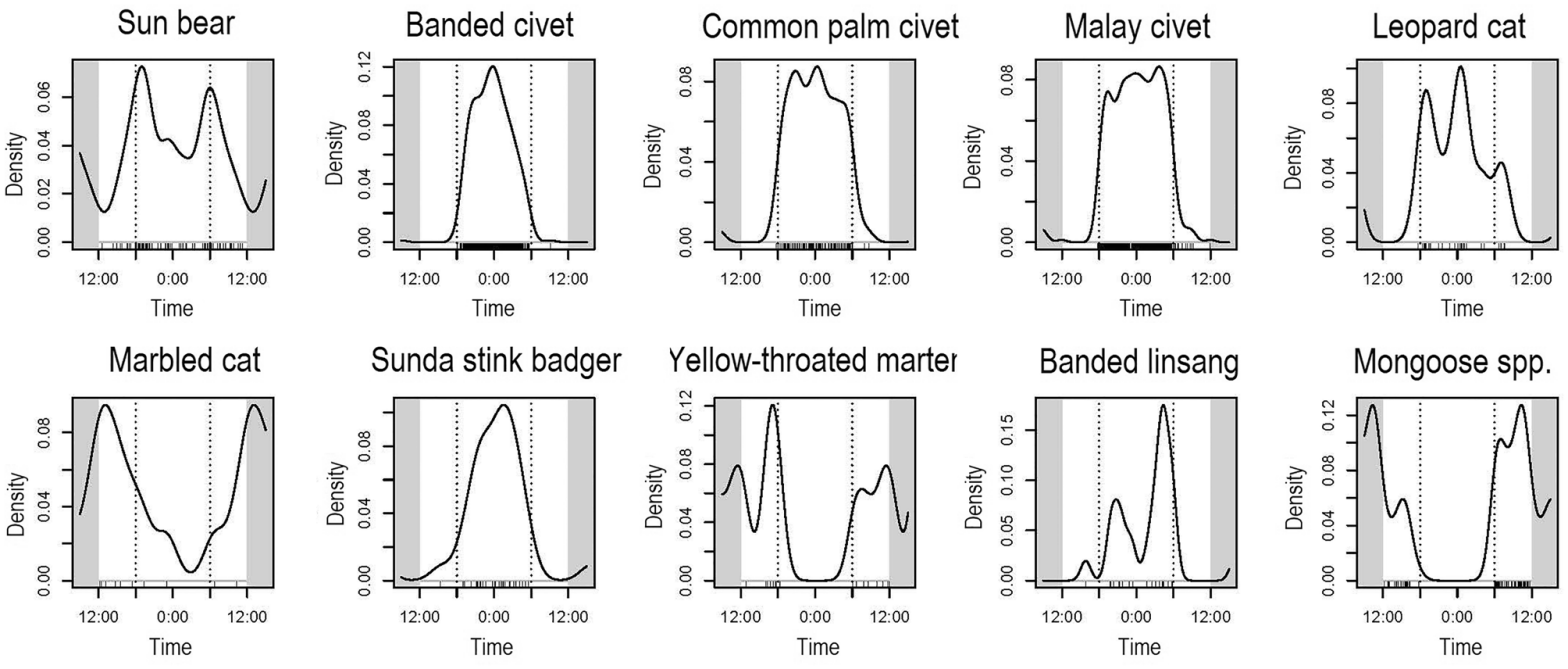
Temporal activity patterns of the ten carnivore species estimated by kernel density estimates. Dotted vertical lines indicate approximate times of sunset and sunrise, and short vertical lines under the kernel density curves indicate photo-captured times of each species.

From these data, we determined that Malay civets, banded civets, common palm civets, Sunda stink badgers, leopard cats, and banded linsangs were strongly nocturnal (Fig. 2). Marbled cats were strongly diurnal, and yellow-throated martens were diurnal and crepuscular (Fig. 2). Note that these species were also active during twilight, so the activity pattern here indicates their tendency. The sun bears were cathemeral (Fig. 2).

### Temporal activity overlaps among the carnivore species

**Figure 3:**
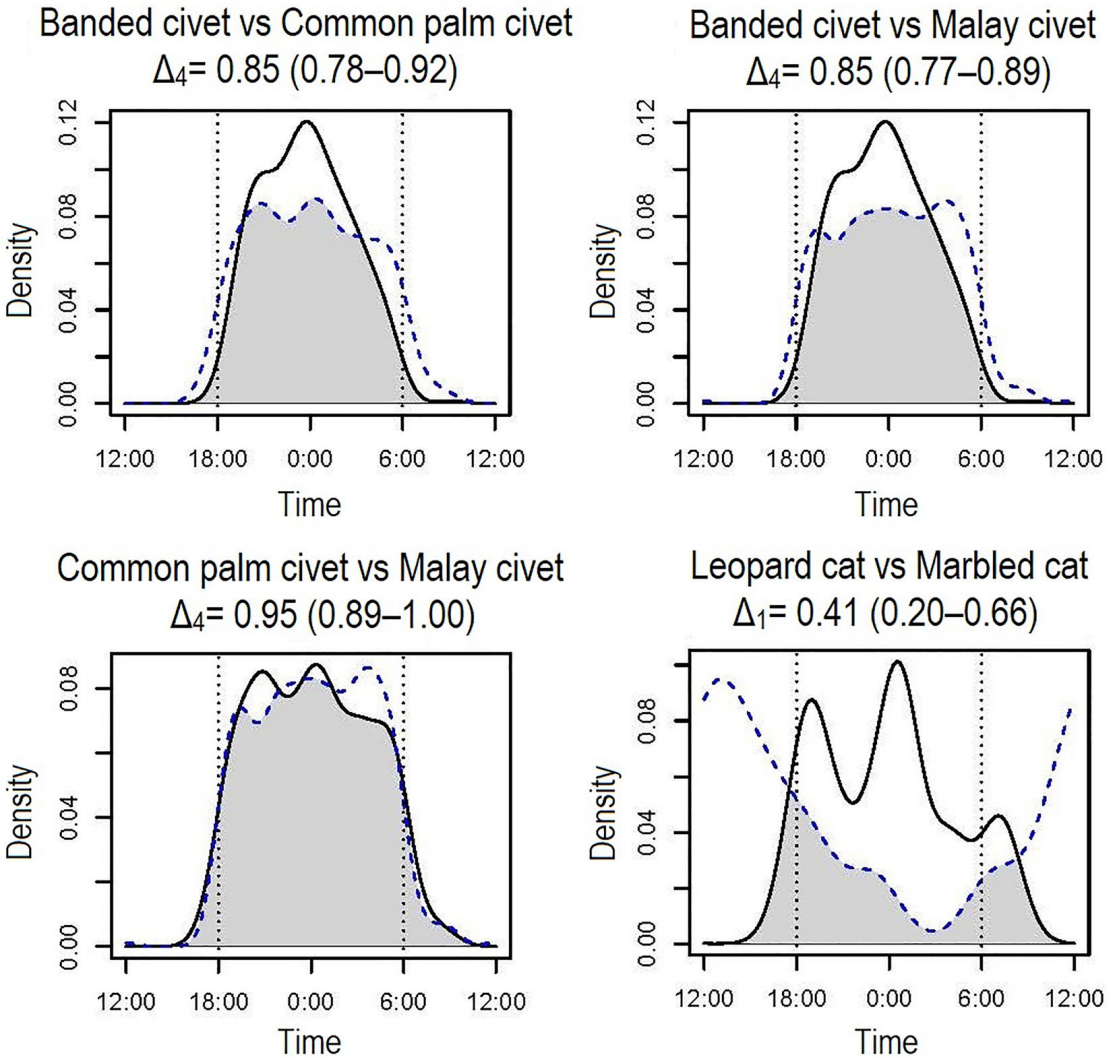
Temporal activity overlaps among the carnivore species belonging to the same family. Solid lines indicate species before "vs", and dotted lines indicate those after "vs". Grey shared areas indicate the coefficient of overlaps (Δ1 and Δ4) of the two density estimates. Dotted vertical lines indicate approximate times of sunset and sunrise, and short vertical lines under the kernel density curves indicate photo-captured times of each species.

The temporal activity overlaps among the analyzed carnivore species are generally high (0.74 ≤ Δ_1_ or Δ_4_) among the nocturnal species (banded civets, common palm civets, Malay civets, Sunda stink badgers, leopard cats), except for banded linsangs, showing moderate values among these species (Δ_1_ < 0.7) (Table 3). Contrary to the nocturnal species, the overlap between diurnal species (marbled cats, yellow-throated martens) was moderate (0.6 < Δ_1_) (Table 3). We found the highest activity overlap between common palm civets and Malay civets (Δ_4_ = 0.95). The level of activity overlaps among species in the same family varied among the taxa; those of civets were highly overlapped (0.85 ≤ Δ_4_), while that of felids (Δ_1_ = 0.41) was low (Table 3, Fig. 3).

**Table 3:** Coefficient of overlaps (Δ_1_ and Δ_4_) of temporal activity patterns between carnivore species.

Family	Species	Compared species							
		Sun bear	Banded civet	Common palm civet	Malay civet	Leopard cat	Marbled cat	Sunda stink badger	Yellow-throated marten
Urusidae	Sun bear	–							
Viverridae	Banded civet	0.56 (0.44–0.63)	–						
	Common palm civet	0.68 (0.58–0.78)	**0.85 (0.78–0.92)**	–					
	Malay civet	0.67 (0.57–0.76)	**0.85 (0.77–0.89)**	**0.95 (0.89–1.00)**	–				
Felidae	Leopard cat	0.73 (0.61–0.89)	**0.74 (059–0.86)**	**0.80 (0.67–0.92)**	**0.79 (0.65–0.89)**	–			
	Marbled cat	0.56 (0.36–0.80)	0.29 (0.06–0.50)	0.35 (0.13–0.58)	0.34 (0.12–0.56)	0.41 (0.20–0.66)	–		
Mephetidae	Sunda stink badger	0.62 (0.44–0.72)	**0.82 (0.73–0.96)**	**0.83 (0.73–0.95)**	**0.84 (0.76–0.95)**	**0.74 (0.58–0.88)**	0.34 (0.11–0.55)	–	
Mustelidae	Yellow-throated marten	0.56 (0.40–0.70)	0.20 (0.03–0.24)	0.29 (0.11–0.37)	0.28 (0.10–0.34)	0.39 (0.20–0.54)	*0.63 (0.45–0.90)*	0.26 (0.05–0.35)	–
Prionodontidae	Banded linsang	0.58 (0.43–0.72)	**0.60 (0.43–0.75)**	**0.67 (0.51–0.84)**	**0.69 (0.53–0.84)**	**0.59 (0.36–0.73)**	0.29 (0.09–0.52)	**0.65 (0.48–0.84)**	0.25 (0.03–0.36)

The values in parenthesis indicate 95% confidence interval. The bold cells indicate overlaps between nocturnal species, and italics indicates those between diurnal species.

## Discussion

In this study, we found that six species (three civets, one skunk, one felid, one linsang) are nocturnal, two species (one felid, one mustelid) and *Mongoose* spp. are diurnal, and one species (bear) is cathemeral in Borneo. We successfully obtained substantial sample sizes of the three civet species (banded civets, common palm civets, Malay civets) and sun bears, and we confirmed that their activity patterns do not differ among the three sites. Therefore, this study would be a thorough reference for the activity patterns of these four species. The results of the activity patterns for the other five species (leopard cats, marbled cats, Sunda stink badgers, yellow-throated martens, and banded linsangs) should be interpreted with caution because we could not distinguish individuals and pooled data from the three study sites, which may have introduced some pseudo-replications^21^. However, given the limited amount of data available on some of these species in general, our data would still contribute to understanding their activity patterns.

Temporal niche partitioning among some species with morphological and/or ecological similarities is observed in this study. First, we found a clear separation of activity patterns between two felid species; leopard cats are strongly nocturnal, while similar-sized marbled cats present diurnal behavior (Table 3, Fig. 3). These results corroborate with the previous study in Sabah^9^. Second, yellow-throated martens and common palm civets on Borneo also have several similarities such as body sizes, diets, and semi-arboreal habits^21^, suggesting that they could be potential competitors, although they belong to different families. However, yellow-throated martens are diurnal and common palm civets are nocturnal, therefore, their temporal activity overlap was low (Table 3), indicating their temporal niche segregation, mitigating negative interactions by avoidance of direct encounters. Contrary to the felids and martens, three civet species of the same family exhibit the most extensive activity overlaps among the observed species (Table 3, Fig. 3). The three civet species have similar diet and body size^22^, and they occur in quite similar spatial and temporal spaces. Although there is no evidence of temporal niche partitioning among the three civet species, there appear to be minor differences in spatial activity patterns among them. Banded civets and common palm civets prefer interior forests, open-canopy habitats such as roadside, respectively, while Malay civets are found in both forest types^23^. These subtle ecological differences would be significant to maintain their coexistence in complex forest structures in Borneo.

Based on our results, yellow-throated martens and *Mongoose* spp. are strictly diurnal, but the other species have nocturnal activity patterns in varying degrees (Table 2, Fig. 2). All three civets, leopard cats, Sunda stink badgers, and banded linsangs are nocturnal, and most of them exhibit high overlaps (0.7 < ∆, Table 3) in their temporal activity patterns, except for banded linsangs. Overall, activity overlaps between banded linsangs and the other nocturnal carnivores are not high (∆_1_ < 0.7, Table 3) compared to the others. During the nighttime, differences in activity peaks may relate to the low activity overlaps of banded linsangs. Banded linsangs show clear bimodal peaks during the night and twilight periods (Fig. 2), and they are most active in the last half of the night (Table 2). Whereas, the other five nocturnal species are active throughout the night, especially in the first half of the night (Table 2). Thus, even among species with the same temporal activity patterns, some species differentiate activity peaks. However, temporal niche overlap among the other five nocturnal species is still quite extensive. A possible reason for their coexistence is dietary niche partitioning. In Borneo, only felids and linsangs are supposed to be hyper-carnivores^22,24^, while the other species are highly omnivorous: feeding on mammals, birds, invertebrates and plant matter^23^. Although information regarding the diets of most Bornean carnivore species is still scarce, such broad dietary breadth may compensate for the high temporal niche overlaps among the nocturnal carnivores.

Most of the studied carnivore species are small to medium (< 10 kg^22^) except for the sun bears. In a guild of five African sympatric small-medium carnivores (< 10 kg), they are separated into two temporal groups: three nocturnal and two diurnal species^25^. In Madagascar carnivores, comprising a single-family Eupleridae have three nocturnal, one diurnal, and one cathemeral species^26^. In a Neotropical small-medium felid guild, there are two are nocturnal, one diurnal, and one cathemeral species^7^. Thus, it is suggested that the number of nocturnal small-medium carnivore species is large across the continent, most likely due to phylogenetic constraints^27^, but that of the Bornean community overwhelms the others. It remains unclear whether the occurrence of these sympatric carnivore species during the same time periods generates negative effects such as intra-guild killing and interference competitions. Given that temporal niche segregation is one of the most effective mechanisms that diminishes competition^5^, the studied carnivore species may not compete intensively, or have relatively small ecological differences that have not yet been investigated.

Currently, camera-trapping is one of the most basic but effective tools for community ecology and conservation planning in mammals^4^. The temporal activity pattern is one of the main data obtained from camera-trapping. Indeed, our data on temporal activity patterns of common palm civets and Malay civets successfully show results similar to radio-tracking in DVCA and TWR^16,28^, where both are predominantly nocturnal, but also show crepuscular behavior. Our results for sun bears are contradictory to those of an intensive study conducted using both individual radio-tracking and camera-trapping in the DVFC for 2 years^29^; radio-tracking suggested that sun bears were diurnal, whereas camera trapping suggested crepuscular and nocturnal patterns. However, our relatively robust dataset showed that sun bears are cathemeral, and also a previous study with 6 years of camera-trapping records indicated that they were crepuscular^19^. Considering that their activity patterns vary at the individual level^29^, the overall activity patterns of several individuals in a certain area may become cathemeral or crepuscular, being active during both daytime and night time, as indicated by this study and the previous study^19^. The lack of long-term empirical data for any taxon would hinder our understanding of its temporal activity pattern, which could consequently divert conservationists from establishing effective protection measures. Although the photo-capture frequency differed by species and sites, the cumulative number of photos of four species reached 10, which was a sufficient sample size for analyses in this study, after the second (sun bears) and third year (three civet species) (Supplementary Fig. S1 online). When the number of working cameras was less than 10, the slope of curves of the cumulative photo numbers tended to be gentler (Supplementary Fig. S1 online). In terms of species number, the cumulative species numbers saturated after 20, 26, and 24 months in DVCA, LKWS, and TWR, respectively (Supplementary Fig. S2 online). Therefore, we propose that at least 2 or 3 years of long-term study with at least ten cameras is necessary, especially for sun bears and civets, to understand an animal’s temporal activity patterns by camera trapping.

All the study sites are protected areas, but evidence of poaching have been reported, including sun bears in some of these areas^30^. Our results do not show statistical differences in temporal activity patterns of sun bears and the three civet species among the study sites, but this may change depending on the threat status given that some animals change temporal activity patterns because of hunting disturbances^31^. Non-lethal tourism activities may also affect animal activity. Tourism activity was conducted at all study sites during the study period. The potential benefits gained from ecotourism may frequently counteract the risks of exposure to changes in animal activity patterns^32^. In LKWS, community-based ecotourism is common and can bring significant benefits such as alternative income that incentivizes local communities and policy makers to protect the species in areas of interest. Spotlighting activities along the Menanggul River were often conducted by several motor boats during early morning, late afternoon, and night in LKWS. However, no nocturnal tourism activities were conducted around the camera stations in DVCA and TWR. Common palm civets show at least two clear peaks of temporal activity levels in the latter two sites, whereas those in LKWS are unclear and delayed (Fig. 1). Given that common palm civets prefer open-canopy areas including riverine forests^33^, they might be directly affected by tourism activity, especially during nighttime in LKWS. Thus, there may be a need for evaluating the effect of tourism activity on animal behavior in future studies, even though it is non-lethal ecotourism.

Lastly, many studies are using camera-trapping data, including remote areas with relatively poor accessibility. Thus, it is the time to accumulate the information on rare species to determine their basic ecology, including temporal activity patterns and habitat selection, and to reassess the propriety of current conservation management strategies.

## Materials and methods

### Study sites

**Figure 4:**
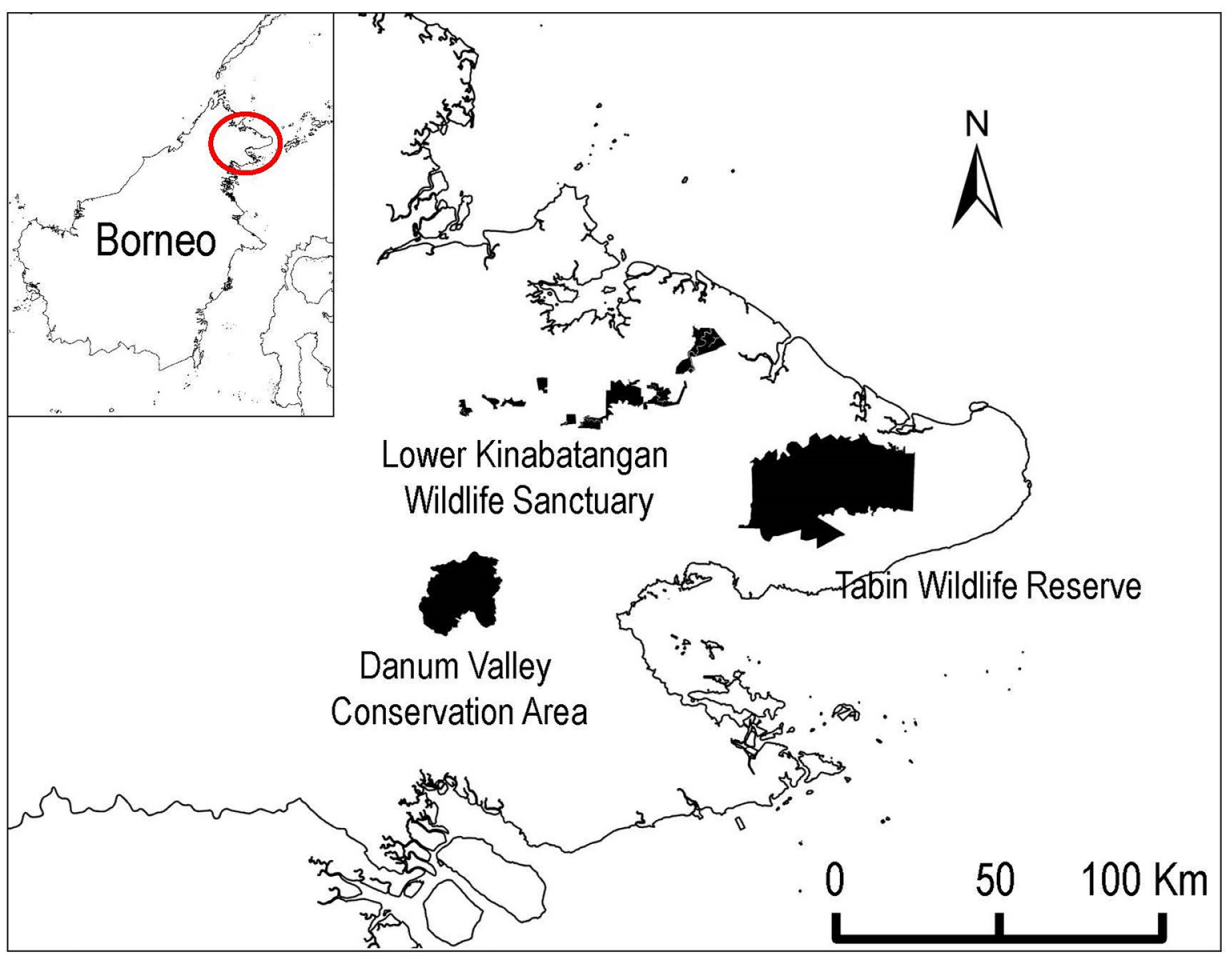
Location of the three study sites in Borneo.

We conducted this study in three protected areas in Sabah, Malaysian Borneo: Danum Valley Conservation Area (DVCA), the Lower Kinabatangan Wildlife Sanctuary (LKWS), and Tabin Wildlife Reserve (TWR) (Fig. 4). The minimum and maximum daily temperatures and annual precipitation among the three study sites did not differ significantly (annual temperature: 22–33 ℃, annual precipitation 2400–3100 mm; Mitchell^37^; Matsuda et al.^39^; South East Asia Rainforest Research Partnership Unpublished data. https://www.searrp.org/) although there is no recent precise climate data of TWR.

The DVCA (4° 50′–5° 05′ N, 117° 30′–117° 48′ E) is a Class I Protection Forest Reserve established by the Sabah state government in 1996 and managed by the Sabah Foundation (Yayasan Sabah Group) covering 438 km^2^. Approximately 90% of the area is comprised of mature lowland evergreen dipterocarp forests^34^. The study area is an old-growth forest surrounding the Borneo Rainforest Lodge (5° 01′ N, 117° 44′ E), a tourist lodging facility.

The LKWS (5° 10′–5° 50′ N, 117° 40′–118° 30′ E), is located along the Kinabatangan River, which is the longest river flowing to the east coast, reaching 560 km inland and with a catchment area of 16,800 km^2^. Designated as a wildlife sanctuary and gazetted in 2005, the LKWS consists of ten forest blocks totaling 270 km^2^, comprised of seasonal and tidal swamp forests, permanent freshwater swamps, mangrove forests, and lowland dipterocarp forests^35,36^. The southern area of the Menanggul River is extensively covered by secondary forest. However, the northern area has been deforested for oil palm (*Elaeis guineensis*) plantations, except for a protected zone along the river. The TWR (5° 05′–5° 22′ N, 118° 30′–118° 55′ E) is located approximately 50 km northeast of Lahad Datu, eastern Sabah, and covers approximately 1225 km^2^.

The TWR is exclusively surrounded by large oil palm plantations. Most parts of the TWR were heavily logged in the 1970s and the 1980s, leaving mainly regenerating mixed dipterocarp tropical rainforests dominated by pioneer species such as *Neolamarckia cadamba* and *Macaranga bancana*^37,38^. The study area was near the Sabah Wildlife Department base camp located on the western boundary of the TWR (5° 11′ N, 118° 30′ E). The study area includes heavily logged secondary forests and a small patchy old forest (0.74 km^2^).

### Data collection

We set up 15, 30, and 28 infrared-triggered sensor cameras (Bushnell, Trophy Cam TM) in the DVCA (July 2010–August 2011 and May 2014–December 2016), LKWS (July 2010–December 2014) and TWR (May 2010–June 2012), respectively. As a result, the cumulative number of camera operation days in DVCA, LKWS, and TWR were 14,134, 18,265, and 4980, for a total of 37,379 days. Although it was impossible to record the animals during certain months because of adverse weather conditions, such as heavy rain, flooding, battery failure, other malfunctions mainly caused by insects nesting inside the cameras, or logistical problems, the cameras remained continuously activated. Due to these reasons, camera operating days differed among the cameras in each site. In this study, we used photos of animals, and we did not handle animals directly. All cameras were placed at heights of 30–50 cm above the forest floor and were tied to tree trunks using fabric belts to reduce damage to the trees.

**Figure 5:**
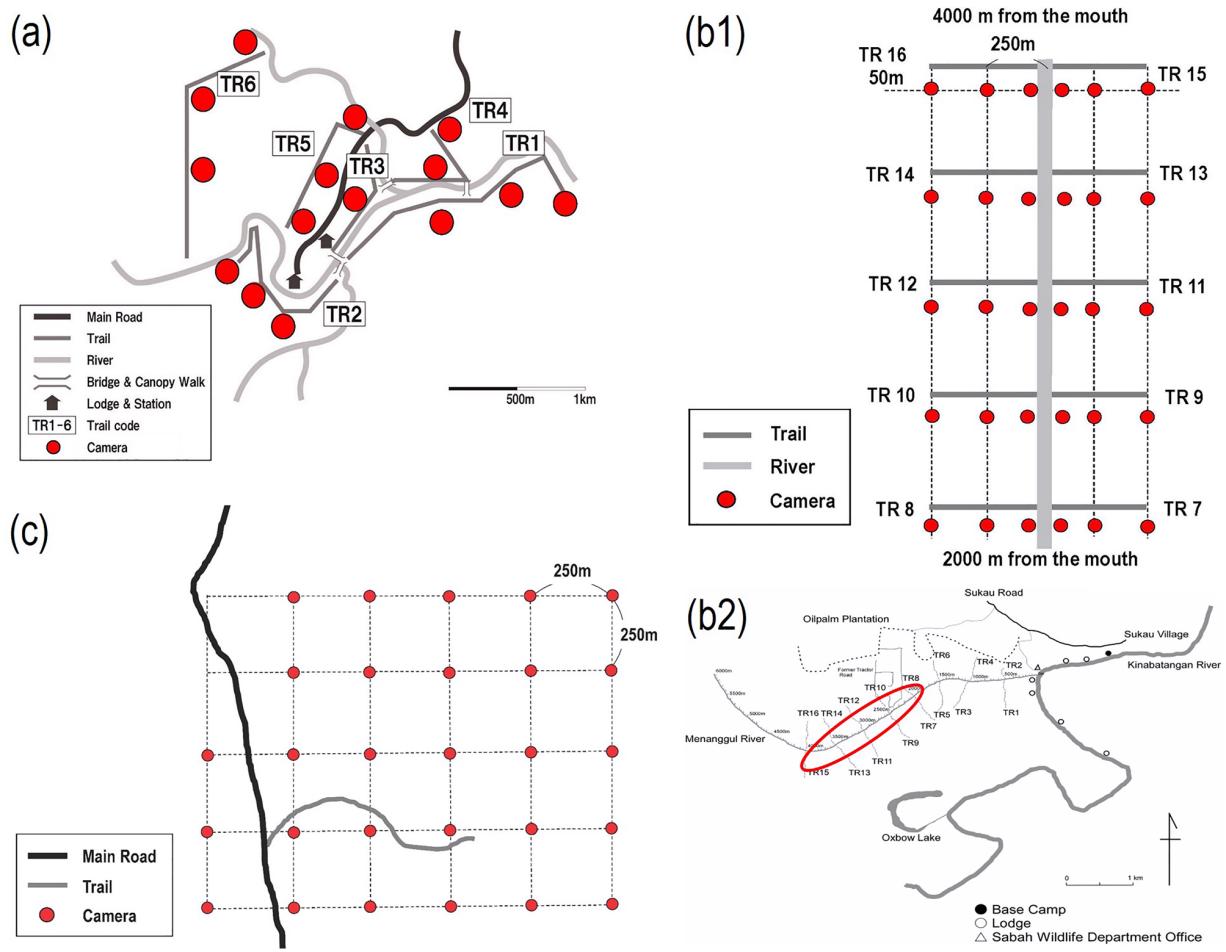
Maps of camera locations at each study site. (**a**) Trails and camera stations at DVCA; (**b1**) trails and camera stations and (**b2**) trail locations at LKWS; (**c**) a trail and camera stations at TWR.

Because the terrain and level of regulations to conduct this study differed by the study site, we employed different layouts of camera stations at each study site. In the DVCA, T. K. and three trained assistants placed 15 cameras along six forest trails totaling 9000 m, which were established and maintained by the tourist lodging facility. Because it was prohibited to establish new trails and to place cameras at sites where tourism activity would be disturbed in the study area; therefore, the trails that were longer than 1 km and relatively easily accessible were selected as camera locations to maintain consistency of trail characteristics. Cameras were placed on each trail at 50 m intervals, alternating right and left to avoid bias of photo-capture frequency caused by terrain differences. Each station was at least 25 m away from each other on the different trails (Fig. 5a). The operating days differed among the 15 cameras, i.e., mean = 942.2; SD = 152.0; range = 682–1229.

In the LKWS, I. M. and two trained assistants had planned to install 30 cameras, but a maximum of only 27 cameras were in operation during the study period in the LKWS, probably owing to malfunctions caused by high humidity and rain in the tropical rainforest. All cameras were placed on the trails in the riverine forest along the Menanggul River. As part of a project on the primates of the riverine forests along the Menanggul River and to assist their observation and tracking in the swampy habitat in the LKWS^39^, trails 200–500 m long and 1 m wide were established at 500 m intervals on both sides of the river. Of the 16 trails, we selected ten trails that were all 500 m long and placed three cameras at the points from the riverbank to the inland forest in each trail, that is, 10 m, 250 m and 500 m from the riverbank (Fig. 5b1); cameras were set up 50 m away from the trails (Fig. 5b2). Consequently, the number of operating days differed among 30 cameras, i.e., mean = 608.8; SD = 531.4; range = 28–1315.

In the TWR, M. N. and A. M placed 28 and three cameras on camera stations created by overlaying a 750 × 500 m grid in May and August 2010, respectively. Cameras were placed at each grid point at 250 m intervals (Fig. 5c). The operating days differed significantly among the 28 cameras, that is, mean = 177.9; SD = 123.2; range = 26–539.

### Temporal activity analysis

We defined non-independent photo capture events as consecutive photos of the same or different individuals of the same species taken within a 30-min interval and removed these photos from the analysis. We plotted the activity patterns of each species using a von Mises kernel^40,41^ using the package activity^42^ in R version 4.0.2^43^. We estimated the activity level of animals with more than ten independent photo-capture events as indicated in the previous studies^26,44^. For our analysis, we pooled the images from all study sites if the photo number of a species was less than 10 in any study locations. If that was not the case, we used the package activity^42^ to compare species activity levels across the three research sites using a Wald test with Bonferroni correction for multiple pairwise comparisons. When there were significant differences, we separately estimated activity levels by the study sites. When there were no significant differences among the sites, we pooled the photo numbers to estimate activity levels.

We divided a day into three periods: nighttime (19:00–04:59 h local time (GMT + 8)); daytime (07:00–16:59 h); and twilight (05:00–06:59 h and 17:00–18:59 h). During the study period, twilight hours essentially corresponded to 1 h between sunset and sunrise, at 5:54–6:25 and 17:50–18:25 in DVCA, 5:51–6:23 and 17:47–18:25 in LKWS, and 5:50–6:21 and 17:46–18:22 in TWR (data from https://www.timeanddate.com). After converting the time data of each photo-capture event into radians, we fitted a circular kernel density distribution estimated by 10,000 bootstrap resampling to radian time data, and we estimated the percentage of active time in each period. We then categorized the activity patterns of photo-captured carnivore species into four categories: nocturnal (active at night); crepuscular (active during twilight periods); diurnal (active during daytime); and cathemeral (active in all periods). We defined the activity pattern of the species as showing a statistically higher proportion of photo-captures at nighttime, daytime, and twilight periods than at other periods, such as nocturnal, diurnal, and crepuscular, respectively. When photo-capture proportions showed no differences among the three periods, we defined the activity pattern as cathemeral. For species with substantial sample size (50 <), we compared the number of independent photo-capture event among the three periods by species using generalized liner mixed models (GLMMs) to determine the activity patterns of each species using the lme4^45^ and multcomp^46^ packages. We set the period as a fixed effect, the study site and the camera position as random effects, and the camera working days as an offset term. For other species, we tested if the animals were selectively active; in other words, they were photo-captured disproportionally during any of the three periods using the package adehabitatHS in R^47^. It was impossible to identify individuals from the photo data when only part of the body was recorded; therefore, we used a design I resource selection function, selecting at the population level^48^.

After these procedures, we evaluated temporal activity overlaps among the species by the coefficients of overlap (Δ) for each species, ranging from 0 (no overlap) to 1 (complete overlap) using the overlap package in R^49^. We used Δ_1_ to estimate the nonparametric overlap coefficient of species with < 75 sample sizes, while we used the Δ_4_ estimator for other species with > 75 photos^40^. Next, we categorized the temporal activity overlap level as: low, moderate, and high, based on the values of coefficients of overlap (Δ) generated by the pairwise comparisons. Low, moderate, and high overlaps indicated that Δ values were ≤ 50, 50 < ∆ ≤ 75, and ∆ > 75, respectively^8^. Finally, we calculated the 95% confidence intervals of the overlap coefficient using a smoothed bootstrap with 10,000 resamples.

## Supplementary Information

Supplementary Information.

Supplementary Figures.

## Data Availability

The data used in this study was included in Supplementary Information.
