# Supplementary material for: Temporal activity patterns suggesting niche partitioning of sympatric carnivores in Borneo, Malaysia
Source: Sci Rep. 2021 Oct 6;11:19819. doi: 10.1038/s41598-021-99341-6 (PMC8494825; doi:10.1038/s41598-021-99341-6)
Supplement: Supplementary file 2 — Supplementary Figures. [file 41598_2021_99341_MOESM2_ESM.pdf]

Temporal activity patterns suggesting niche partitioning of sympatric carnivores in Borneo, Malaysia

Miyabi Nakabayashi[1*], Tomoko Kanamori[2,3], Aoi Matsukawa[4], Joseph Tangah[5], Augustine Tuuga[6], Titol Peter Malim[6†], Henry Bernard[7], Abdul Hamid Ahmad[8], Ikki Matsuda[4,7,9,10], Goro Hanya[3]

[1]Graduate School of Advanced Science and Engineering, Hiroshima University, Hiroshima, Japan

[2]Japan Orangutan Research Center, Tokyo, Japan

[3]Primate Research Institute, Kyoto University, Aichi, Japan

[4]Wildlife Research Center, Kyoto University, Kyoto, Japan

[5]Forest Research Centre, Sabah Forestry Department, Sandakan, Sabah, Malaysia

[6]Sabah Wildlife Department, Kota Kinabalu, Sabah, Malaysia

[†] Titol Peter Malim passed away on 6th February 2021. His contributions as an author are listed in the Author Contributions section.

[7]Primate Studies-Borneo, Institute for Tropical Biology and Conservation, Universiti Malaysia Sabah, Sabah, Malaysia

[8]Institute for Tropical Biology and Conservation, Universiti Malaysia Sabah, Sabah, Malaysia

[9]Chubu University Academy of Emerging Sciences, Aichi, Japan

[10]Japan Monkey Centre, Aichi, Japan

[*]Corresponding author

E-mail: miyabi.nakabayashi@gmail.com

Phone number: +81-82-424-6930

Supplementary Figures

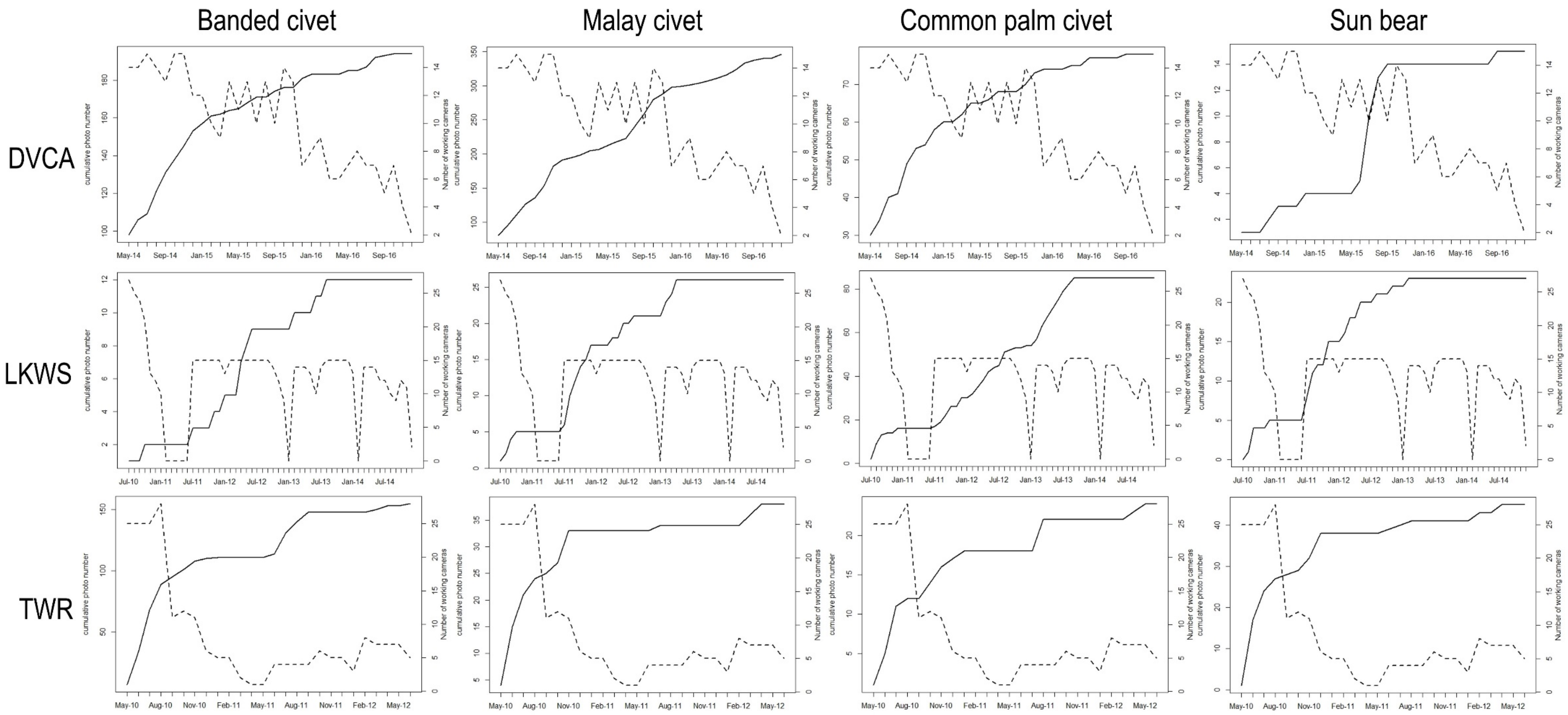

Supplementary Figure S1 Monthly numbers of cumulative photos of four carnivore species (banded civet, Malay civet, common palm civet and sun bear) and working cameras in the (a) DVCA, (b) LKWS, and (c) TWR. Dotted line and solid line indicate number of working cameras and cumulative photo number, respectively. Note that durations of more than three consecutive years are shown.

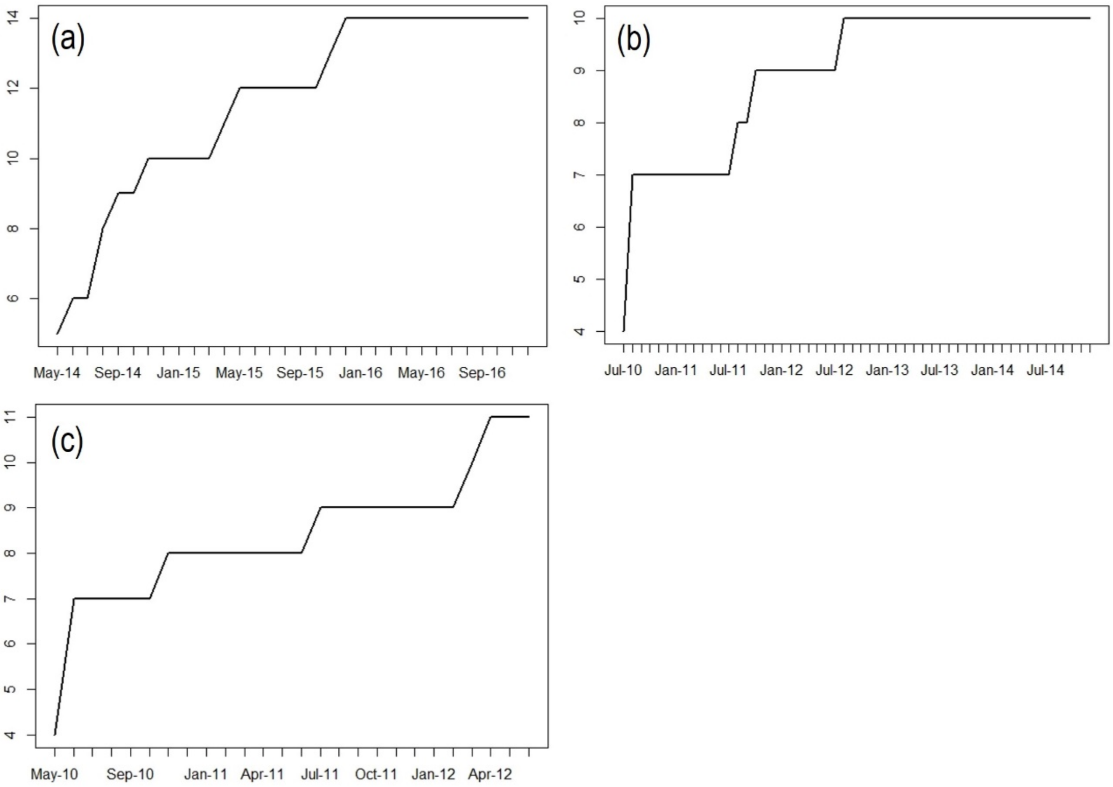

Supplementary Figure S2 Monthly numbers of cumulative species of carnivores in the (a) DVCA, (b) LKWS, and (c) TWR. Note that durations of more than three consecutive years are shown.
